# Health-Related Quality of Life and Its Influencing Factors in Patients with Hepatitis B: A Cross-Sectional Assessment in Southeastern China

**DOI:** 10.1155/2021/9937591

**Published:** 2021-07-07

**Authors:** Ping Chen, Fen Zhang, Yiqun Shen, Yubo Cai, Chaolei Jin, Yan Li, Mingmin Tu, Weizhen Zhang, Yu Wang, Shi-Feng Zhang, Jiangyun Wang, Lanjuan Li

**Affiliations:** ^1^Department of Infectious Diseases, Shulan (Hangzhou) Hospital Affiliated to Zhejiang Shuren University Shulan International Medical College, Hangzhou 310012, China; ^2^Department of Infectious Diseases, Zhengzhou Cancer Hospital of Henan University, Zhengzhou 450000, China; ^3^State Key Laboratory for Diagnosis and Treatment of Infectious Diseases, National Clinical Research Center for Infectious Diseases, Collaborative Innovation Center for Diagnosis and Treatment of Infectious Diseases, The First Affiliated Hospital, College of Medicine, Zhejiang University, Hangzhou 310003, China; ^4^Huzhou Central Hospital, Affiliated Central Hospital Huzhou University, HuZhou 313000, China; ^5^School of Medicine, Zhejiang University, Hangzhou 310058, China; ^6^School of Clinical Medicine, Henan University, Kaifeng 475000, China; ^7^The Academician Workstation, Zhengzhou Cancer Hospital of Henan University, Zhengzhou 450000, China; ^8^Hangzhou Tongchuang Medical Laboratory, Hangzhou 310012, China; ^9^Department of Oncology, Zhengzhou Cancer Hospital of Henan University, Zhengzhou 450000, China; ^10^Department of Hematology, Zhengzhou Cancer Hospital of Henan University, Zhengzhou 450000, China; ^11^Department of Stomatology, Zhengzhou Cancer Hospital of Henan University, Zhengzhou 450000, China; ^12^Department of Cosmetic Surgery, Zhengzhou Cancer Hospital of Henan University, Zhengzhou 450000, China

## Abstract

Health-related quality of life (HRQoL) is an important aspect in the management of patients with hepatitis B (HB), which remains a serious health problem in China. There have been relatively few HRQoL studies involving Chinese patients with HB. The aim of this study was to analyze HRQoL in patients diagnosed with HB living in Zhejiang Province, China. A cross-sectional sample of 98 patients with chronic HB (CHB), 56 patients with advanced HB that have developed cirrhosis, and 48 healthy controls (HCs), all from Zhejiang Province, was used in this study. HRQoL was assessed using Short-Form 36 (SF-36) version 2, European quality of life questionnaire-5 dimensions (EQ-5D), and chronic liver disease questionnaire (CLDQ). Intergroup score differences were detected with *U* tests. Factors with a significant effect on HRQoL were identified with Spearman correlational analyses. Patients with HB (both groups) had lower SF-36 scores than HCs (*p* < 0.01), with the exception of general health subscores. Patients with HB cirrhosis had the lowest scores in the EQ-5D visual analog scale (VAS) component. Furthermore, patients with HB cirrhosis had lower (*p* < 0.01) CLDQ scores than patients with CHB. In our HB patient cohort, disease stage and income level were the factors most associated with HRQoL variables; age, education level, and marital status were, each, also significantly associated with some HRQoL variables in patients with HB in our study (*p* < 0.05 or *p* < 0.01). HRQoL is diminished in patients with HB in southeastern China. Disease stage and income emerged as key determinants of HRQoL scores. Augmenting social and medical supports for patients with HB, especially those with a socioeconomic status and an advanced disease stage, may help to enhance HRQoL.

## 1. Introduction

Hepatitis B (HB) is a serious and incurable disease caused by infection with HB virus (HBV) [1]. HBV infection can be confirmed with HB surface antigen (HBsAg) testing. According to a recent global hepatitis report produced by the World Health Organization, 240 million individuals were HBsAg-positive worldwide in 2017, including more than 74 million in China alone [2,3]. Hence, the geographical distribution of people infected with the HBV is quite unbalanced, with the largest portion of affected people living in China [4,5]. Additionally, of the approximately 850,000 US citizens and residents reported in 2012 to have been diagnosed with chronic HB (CHB), nearly half were of non-Hispanic Asian descent [4].

Newborn HBV vaccination was introduced in China in 1992 [6–8]. In 1992, nearly one in ten people in the mainland Chinese population (9.75%) were HBsAg-positive; by 2006, after 14 years of intensive vaccination, the HBsAg-positive rate had dropped to 7.18% [6]. Hence, though infection rates are improving, the HBV-infected population in China remains fairly high, and HBV is an ongoing public health challenge. It has been suggested that vaccination schedule delays are an important factor in the slow progress in HBV-infection rate reduction in China [9].

Patients with CHB exhibit a variety of disease processes, including liver decompensation, cirrhosis, and even primary hepatocellular carcinoma, which can severely affect the quality of life and life expectancy. The Global Health Data Exchange data for 2017 showed a loss of about 21 million disability-adjusted life-years due to HB-related morbidity [10]. Although clinically available antiviral treatments can delay CHB disease progression and improve outcomes for patients diagnosed with CHB, high treatment costs can have severe degradative effects on patients' quality of life [11], with more than 10,000 Yuan per year being spent on drugs and routine examinations for each patient in China [10]. Since chronic hepatitis C has been shown to affect health-related quality of life (HRQoL) [12,13], there has been an increasing interest in examining the impacts of HB on HRQoL, with numerous studies exploring the relationship between HB and HRQoL in patients living in the USA, Vietnam, Singapore, and Hong Kong having been published [10,14–16]. Data describing HRQoL among patients with HB in China remain scarce, especially for patients in Zhejiang Province.

In this study, we evaluated the HRQoL of patients diagnosed with HB in southeastern China, including patients with CHB as well as patients with cirrhosis secondary to HBV infection. HRQoL was assessed with the Short-Form 36 (SF-36) version 2, the European quality of life questionnaire-5 dimensions (EQ-5D), and the chronic liver disease questionnaire (CLDQ); and the mood (including depressive symptoms) of the patients was further assessed with the SF-36 and EQ-5D. Potential HRQoL-influencing factors in the study population were assessed with a multivariate correlational analysis. The present study is intended to produce data that can improve our understanding of HRQoL among patients with HB in southeastern China and thus provide guidance for improving the provision of health services and timely interventions for this patient population.

## 2. Respondents and Methods

### 2.1. Study Population

This project was funded by the Mega-Project for National Science and Technology Development (as part of the 12^th^ five-year plan for China) and the Department of Health of Zhejiang Province. This study was carried out in 2013 principally in Huzhou City in the northern region of Zhejiang Province, which is located in southeastern China. Huzhou City is of median economic development for Zhejiang Province with a relatively small transient population. We invited the 300 patients with CHB who have been receiving CHB management at Huzhou Central since 2008, including taking antiviral drugs, to participate in this study. Forty-eight HCs were recruited from patients receiving their regular annual physical examinations at Huzhou Central Hospital. All potential participants were asked to sign written informed consent forms. This study was conducted in compliance with the principles of the 1975 Declaration of Helsinki and was approved by the Ethics Committee of the First Affiliated Hospital, Zhejiang University.

CHB diagnoses were made in accordance with the standards established in 2000 by the Chinese National Conference on Viral Hepatitis in Xi'an. Cirrhosis due to HB was diagnosed based on liver biopsy (performed for medical reasons, not for this study) and ultrasonographic evidence of ascites, gastroesophageal varices, hepatic decompensation changes, and/or hepatic encephalopathy. The inclusion criteria for patients with HB were as follows: a diagnosis of CHB without cirrhosis (CHB group) or with cirrhosis (HB cirrhosis group); currently receiving antiviral therapy; willingness to provide informed consent for participation [17]. The exclusion criteria for patients with HB were discontinuation of antiviral therapy; any chronic disease comorbidity; previous or current history of mental illness. The inclusion criteria for HCs were as follows: age in the range of 18–80 years and willingness to provide informed consent for participation [17]. The exclusion criteria for HCs were any chronic disease diagnosis and previous or current history of mental illness.

The 300 potential patients with HB invited to participate in this study all had complete diagnostic records. Of these 300 patients, 146 patients were excluded for not receiving antiviral therapy, leaving 164 patients with HB (see Supplementary Figure 1 for patient enrollment flow summary). All of the 48 HCs recruited met the inclusion criteria and none were excluded due to the exclusion criteria. Thus, we administered questionnaires to the remaining 164 patients and 48 HCs; 10 patients returned incomplete questionnaires and were thus not included in the final analyses. Thus, 154 patients with HB and 48 HCs were included in our final analyses.

### 2.2. Interview Process

Participants' HRQoL levels were assessed by trained interviewers using the SF-36 version 2, EQ-5D, and CLDQ [18]. Simultaneously, a self-designed questionnaire was administered to acquire each participant's sociodemographic information [19–21], including gender, age, education level, occupation, household monthly income, course of illness, current health status, cognitive recognition of HB, and medicinal treatment compliance. Cognitive recognition of HB was evaluated with nine yes-no questions (1 point per question) based on the recommendations of Gallegos-Orozco et al. [22,23] such that scores ≤6 points indicated a lack of disease recognition, 7 points indicated fair recognition, and 8 points represented good or better recognition. Medicinal compliance was evaluated with six yes-no questions (1 point per question), with scores ≥ 4 considered to indicate satisfactory compliance and scores ≤1 indicated poor compliance.

The SF-36 instrument consists of 36 items encompassing the following eight domains [24]: physical function, role physical, bodily pain, general health, role emotional, social function, vitality, and mental health. The first four-dimension subscores were summed to produce a physical status component summary (PCS) score; the latter four-dimension subscores were summed to produce a mental health component summary (MCS) score. These two summary scores were then added to yield an overall HRQoL score, with higher scores indicating an overall better HRQoL.

The EQ-5D questionnaire [24] consists of a health descriptive component and a visual analog scale (VAS) component. The health descriptive component has five dimensions (mobility, self-care, usual activities, pain/discomfort, and anxiety/depression); each dimension was graded according to three levels of severity. We calculated overall EQ-5D-3L scores according to the UK TTO value set (range: −0.594 to 1.0). The EQ-5D VAS is a 20-cm-long, 0–100-point scale, wherein scores indicate health status from the worst imaginable state (score of 0) to the best imaginable state (score of 100).

The CLDQ is a 29-item liver-disease-specific scale composed of six dimensions [25]: abdominal symptoms, fatigue, systemic symptoms, activity, emotional function, and worry. The total CLDQ score is an average of the six-dimensional scores, in which higher scores indicate a better quality of life.

### 2.3. Data Analysis

Pearson's chi-square tests were used to compare sociodemographic variables between HB subjects and HCs. Because a normal distribution test indicated that the data were nonparametric, Kruskal–Wallis tests were used adopted to compare sociodemographic variables among the three groups (CHB, HB cirrhosis, and HC), and Mann–Whitney *U* tests were used to compare SF-36, EQ-5D, and CLDQ scores among the three groups and between the two HB groups. Spearman correlation values were calculated to identify independent factors that are significantly related to HRQoL. The raw data were analyzed in SPSS 25.0 with a significance criterion of *p* < 0.05. Mean (*M*) values are reported with standard deviations (SDs) and 95% confidence intervals (CIs); median values are reported with interquartile ranges.

## 3. Results

### 3.1. Study Group Characteristics

The majority of our sample of patients diagnosed with HB was males (69.5%), with a mean age of 50.59 ± 10.31 years and a mean income level above 1500 per month per family. The sociodemographic characteristics of each group are summarized in Table 1**(**and the patients' biochemical blood test results are summarized by group in Supplementary Table 1**)**. Notably, compared with HCs, the CHB group was more likely to be older, males, and nonprofessionals and had a lower income. Patient compliance levels were fair, with about 85% of the patients complying with their doctors' instructions overall. The majority of patients with HB had a good cognitive understanding of their disease, while only about one in seven patients with HB had a poor understanding. The HB cirrhosis group exhibited significantly better treatment compliance than the CHB group (*p* < 0.05). Nearly 30% of patients in the HB cirrhosis group reported that they were less than two years into their disease course, which suggests that many may have been unaware of their infection and health status for a substantial period of time (Table 1).

### 3.2. HRQoL

#### 3.2.1. SF-36

**Figure 1:**
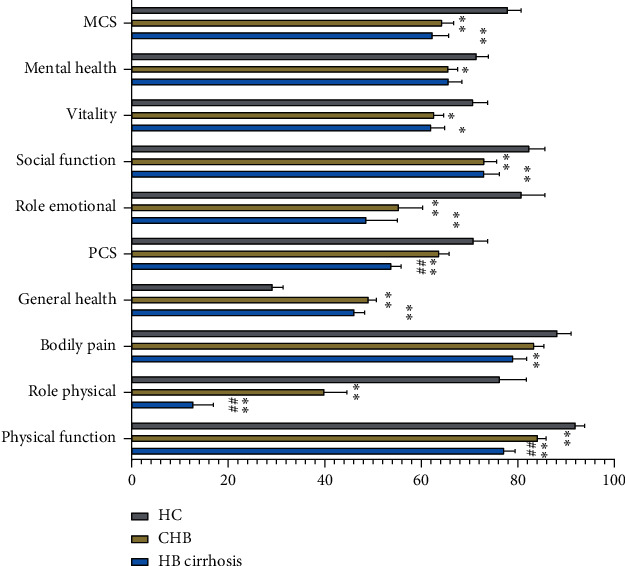
Comparison of SF-36 results across study groups. Note that compared with HCs, the HB cirrhosis group had significantly reduced scores for all dimensions, except for mental health, and the CHB group had significantly reduced scores for all dimensions except for bodily pain and PCS scores. The two HB groups only differed from one another in the role physical and PCS scores. ^*∗*^*p* < 0.05 and ^*∗∗*^*p* < 0.01 vs. HC. ^#^*p* < 0.05 and ^##^*p* < 0.01 vs. CHB.

Compared with HCs, the CHB group had a significantly lower physical function, role physical, role emotional, social function, vitality, and mental health subscores as well as a lower MCS score (Table 2). Meanwhile, compared with HCs, the HB cirrhosis group had a lower physical function, role physical, bodily pain, role emotional, social function, and vitality subscores as well as lower MCS and PCS scores (Table 2). Conversely, compared with the HC group, the CHB and HB cirrhosis groups had higher general health subscores (Figure 1 and Table 2). Compared with the CHB group, the HB cirrhosis group had significantly lower PCS scores but significantly higher physical functioning and role physical subscores (Figure 1 and Table 2).

#### 3.2.2. EQ-5D

**Figure 2:**
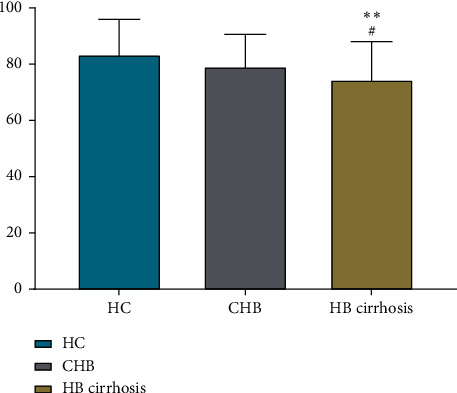
Comparison of EQ-5D VAS scores between study groups. The HB cirrhosis groups had significantly (^*∗∗*^*p* < 0.01) lower VAS scores than the HC group and significantly (^*∗*^*p* < 0.05) lower VAS scores than the CHB group.

There were no significant differences among the three groups in the health descriptive component of the EQ-5D (Table 3). However, patients with HB cirrhosis had slightly lower VAS scores than patients with CHB (75 vs. 79) and markedly lower VAS scores than the HC group (75 vs. 83) (Figure 2).

#### 3.2.3. CLDQ

**Figure 3:**
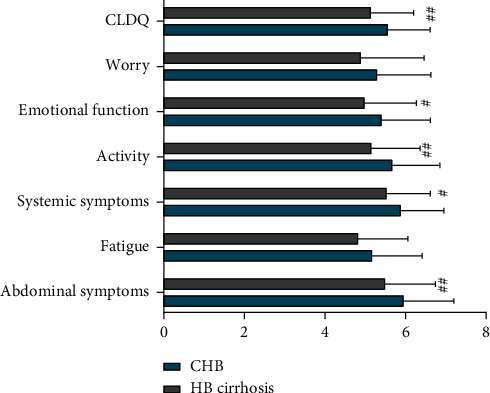
Comparison of CLDQ scores between HB groups. Compared with the CHB group, the HB cirrhosis group had significantly worse (^#^*p* < 0.05) emotional function and systemic symptom scores as well as significantly worse (^##^*p* < 0.05) total CLDQ, activity, and abdominal symptom scores.

The HB cirrhosis group had significantly lower CLDQ scores than the CHB group in the abdominal symptoms, systemic symptoms, activity, and executive function domains of the CLDQ (all *p* < 0.05; Table 4 and Figure 3).

### 3.3. Correlation Analysis

Correlation analyses examining which participant characteristics are associated with HRQoL-representative variables (PCS score, MCS score, SF-36 total score, EQ-D5 VAS score, and CLDQ score) showed that the disease stage, age, education, marital status, and household income were significantly associated with HRQoL. Furthermore, disease stage and household income appeared to be the factors that were most strongly related to HRQoL (Table 5).

Because some respondents did not answer all questions, the number of valid responses for each characteristic may vary.

## 4. Discussion

In this study, we observed broadly lower HRQoL scores in patients with HB, especially patients with HB cirrhosis, compared with HCs. However, the patients with HB, surprisingly, had higher general health SF-36 subscores than HCs in our study. This general health finding is inconsistent with the results of several previous reports showing an opposite trend [16,24,26] Meanwhile, Thuluvath et al. [13] and Helbling et al. [11] found no significant difference in SF-36 general health subscores between patients with HB and HCs.

Our findings of lower MCS scores for the CHB and HB cirrhosis groups, compared with HCs, suggest that the mental health of patients with HB should be further considered. Although the prevalence of major depressive disorder in China is only 1.5%, the prevalence of major depressive disorder among Chinese patients with HB has been reported to be 6.4% [27,28]. In addition to the physical challenges faced by patients with HB, which may include fatigue, appetite loss, and abdominal pain, the disease itself may result in patients having to endure isolation and even discrimination. In this context, it has been suggested that poor self-awareness may contribute to depression in patients suffering from CHB [28].

The present EQ-5D results showing significant intergroup differences for only the VAS component of the instrument are in line with Ong et al.'s findings [16]. Patients with HB cirrhosis had lower CLDQ scores for most of the dimensions, with the exceptions being the fatigue and worry dimensions. Mental health variable scores were similar between the CHB and HB cirrhosis groups, although the latter group had significantly worse bodily pain and PCS scores on the SF-36 than HCs (Table 2), while the CHB group did not—they would be expected to experience a greater hardship as a result of their condition. The lack of a significant difference between these two HB groups with respect to mental health could reflect some level of adaptation to the diminished state in patients with HB cirrhosis, including having reduced expectations regarding the quality of life [14,29]. Based on these results, it is our view that patients with HBV do not often receive sufficient physical and mental health support.

Our correlational analysis performed to identify HRQoL determinants in patients with HB showed that HRQoL was inversely related to an advanced disease stage, older age, being without a life partner (unmarried or separated), low education, and lower income levels, consistent with the findings of several prior studies [10, 12, 15, 29]. The variables of disease stage and household monthly income emerged as the factors most strongly related to diminution of HRQoL. Thus, our data suggest that these two factors may be key influencing factors of HRQoL. Because household income is related to many other sociodemographic variables, including occupation, education level, and marital status, as well as one's understanding of their disease, it may be a useful composite index for HRQoL in patients suffering from a chronic disease, such as HB. Parkash et al. have argued that a low income status due to a limited education is likely an important reason for delays in patients receiving disease diagnoses and treatment; such delays, consequently, result in patients' disease conditions having a more profoundly negative influence on the quality of life [30]. These findings underscore the need for economic analyses of newly approved treatment regimens and those in the clinical trial pipeline, as well as the need for the development of cost-effective strategies. Additionally, our findings indicate that there are unmet needs with respect to targeted services and timely clinical interventions for HB patient populations at a high risk of a low HRQoL.

The current study had several noteworthy limitations. First, we had a relatively small sample size, which increases the risk of type II errors in our statistical analyses. Additionally, the small sample size also precluded us from further dividing our patients according to liver function level. Second, the study population was not fully representative of all patients with HB in Zhejiang Province. Finally, the study sample was somewhat heterogeneous in that participants were recruited from several hospitals in several cities, which can introduce noise into the results.

In conclusion, we documented diminished HRQoL levels in patients with HB in southeastern China. We observed particularly strong significant inverse associations of HRQoL with disease stage and income level in patients with HB, as well as significant associations with older age, being without a marital partner, and education level. Based on these findings, it is our view that more social and medical supports are needed for patients with HB in southeastern China, particularly those with an advanced disease stage and low household income, to improve HRQoL in this population.

## Figures and Tables

**Table 1:** Sociodemographic characteristics and clinical parameters of the respondents (because some respondents did not answer all questions, the numbers of valid responses for characteristics differ).

Characteristic		HC		CHB		HB cirrhosis	
		*N*	%	*N*	%	*N*	%
Gender^*∗∗*^	Male	22	50.0	68	69.4	39	69.6
	Female	22	50.0	30	30.6	17	30.4

Age^*∗∗*##^	<40 years	29	64.4	20	20.4	3	5.4
	40–50 years	10	22.2	37	37.8	15	26.8
	50–60 years	2	4.4	28	28.6	28	50.0
	≥60 years	4	8.9	13	13.3	10	17.9

Education^*∗∗*##^	Elementary school or less	2	4.3	45	45.9	35	62.5
	Middle/high school	27	58.7	48	49.0	21	37.5
	College/postgraduate	17	37.0	5	5.1	0	0.0

Marital status^*∗∗*^	Unmarried	13	28.3	6	6.1	0	0.0
	Married	28	60.9	86	87.8	52	92.9
	Separated	5	10.9	6	6.1	4	7.1

Household monthly income/person^*∗∗*#^	<1500 yuan	4	9.1	28	28.6	21	37.5
	1500–3000 yuan	3	6.8	27	27.6	17	30.4
	≥3000 yuan	37	84.1	43	43.9	18	32.1

Compliance^#^	Poor	—	—	13	13.4	1	1.9
	Medium	—	—	20	20.6	14	25.9
	Good	—	—	64	66.0	39	72.2

Cognitive recognition of disease	Low	—	—	14	14.3	8	14.3
	Medium	—	—	22	22.4	17	30.4
	High	—	—	62	63.3	31	55.4

Disease course	≤2 years	—	—	28	28.6	16	29.1
	3–7 years	—	—	34	34.7	21	38.2
	≥7 years	—	—	36	36.7	18	32.7

^*∗*^ *p* < 0.05 and ^*∗∗*^*p* < 0.01 HB (both groups) vs. HCs. ^#^*p* < 0.05 and ^##^*p* < 0.01 CHB vs. HB cirrhosis.

**Table 2:** Comparison of SF-36 results by group.

SF-36 domain or section summary	HC		CHB		HB cirrhosis	
	Mean (SD)	95% CI	Mean (SD)	95% CI	Mean (SD)	95% CI
Physical function	92.16 (1.7)	88.74–95.57	84.37 (1.52)^*∗∗*^	81.35–87.39	77.35 (2.1)^*∗∗*##^	73.14–81.56
Role physical	76.47 (5.3)	65.82–87.13	40.1 (4.51)^*∗∗*^	31.15–49.06	12.95 (3.98)^*∗∗*##^	4.97–20.92
Bodily pain	88.39 (2.66)	83.04–93.74	83.57 (1.86)	79.87–87.27	79.21 (2.64)^*∗∗*^	73.93–84.5
General health	29.41 (1.97)	25.44–33.37	49.29 (1.41)^*∗∗*^	46.49–52.09	46.34 (1.93)^*∗∗*^	42.47–50.2
PCS	71.06 (2.69)	65.63–76.48	63.89 (1.91)	60.1–67.68	53.96 (1.89)^*∗∗*##^	50.17–57.75
Role emotional	80.95 (4.66)	71.58–90.33	55.56 (4.75)^*∗∗*^	46.12–64.99	48.81 (6.24)^*∗∗*^	36.3–61.31
Social function	82.57 (3.05)	76.44–88.7	73.24 (2.4)^*∗∗*^	68.48–78	73.21 (2.97)^*∗∗*^	67.27–79.16
Vitality	70.92 (2.84)	65.21–76.62	62.85 (1.78)^*∗*^	59.31–66.39	62.23 (2.63)^*∗*^	56.97–67.5
Mental health	71.67 (2.23)	67.19–76.15	65.8 (1.72)^*∗*^	62.39–69.22	65.86 (2.56)	60.72–70.99
MCS	78.14 (2.57)	72.96–83.33	64.49 (2.24)^*∗∗*^	60.05–68.93	62.53 (3.15)^*∗∗*^	56.22–68.83

^*∗*^ *p* < 0.05 and ^*∗∗*^*p* < 0.01 vs. HC; ^##^*p* < 0.01 vs. CHB.

**Table 3:** Comparison of EQ-5D dimension score levels and VAS scores between study groups.

Component Dimension: level		HC		CHB		HB cirrhosis	
		*N*/*M*	%/95% CI	*N*/*M*	%/95% CI	*N*/*M*	%/95% CI
Mobility	Good	46	95.8	96	98.0	54	96.4
	Medium	2	4.2	2	2.0	2	3.6
	Serious	0	0.0	0	0.0	0	0.0

Self-care	Good	46	95.8	96	98.0	56	100.0
	Medium	2	4.2	2	2.0	0	0.0
	Serious	0	0.0	0	0.0	0	0.0

Usual activities	Good	45	93.8	91	92.9	52	92.9
	Medium	3	6.3	6	6.1	4	7.1
	Serious	0	1.0	1	1.0	0	0.0

Pain/discomfort	Good	41	85.4	77	79.4	44	78.6
	Medium	6	12.5	20	20.6	12	21.4
	Serious	1	2.1	0	0.0	0	0.0

Anxiety/depression	Good	38	80.9	76	78.4	41	73.2
	Medium	7	14.9	20	20.6	14	25.0
	Serious	2	4.3	1	1.0	1	1.8

VAS		48 (84)	80–87	98 (79)	77–82	56 (75)^*∗∗#*^	71–78

		Mean	SD	Mean	SD	Mean	SD
EQ-5D-3L		0.94	0.12	0.92	0.13	0.92	0.15

^*∗∗*^ *p* < 0.01 vs. HC; ^#^*p* < 0.05 vs. CHB.

**Table 4:** Comparison of CLDQ dimension and total scores between HB groups.

Dimension	CHB		HB cirrhosis	
	mean (SD)	95% CI	mean (SD)	95% CI
Abdominal symptoms	5.95 (1.25)	5.7–6.2	5.49 (1.25)^##^	5.15–5.82
Fatigue	5.17 (1.24)	4.92–5.42	4.82 (1.24)	4.48–5.15
Systemic symptoms	5.88 (1.07)	5.67–6.1	5.53 (1.09)^#^	5.24–5.82
Activity	5.67 (1.18)	5.44–5.91	5.15 (1.21)^##^	4.82–5.47
Emotional function	5.4 (1.22)	5.15–5.65	4.98 (1.29)^#^	4.64–5.33
Worry	5.29 (1.34)	5.02–5.56	4.89 (1.57)	4.46–5.31
All dimensions averaged	5.56 (1.05)	5.35–5.77	5.14 (1.06)^##^	4.86–5.43

^#^ *p* < 0.05 and ^##^*p* < 0.01 vs. CHB.

**Table 5:** Correlation analysis of sociodemographic factor variable influences on HRQoL outcome variables.

HRQoL outcome variable	Correlated sociodemographic factor, correlation index (4)					
	Disease stage	Gender	Age	Education	Marital status	Household monthly income
SF-36	−0.322^*∗∗*^	−0.076	−0.331^*∗∗*^	0.231^*∗∗*^	−0.190^*∗∗*^	0.394^*∗∗*^
PCS	{195}	{190}	{192}	{192}	{194}	{190}
SF-36	−0.234^*∗∗*^	−0.067	−0.172^*∗*^	0.196^*∗∗*^	−0.073	0.416^*∗∗*^
MCS	{193}	{189}	{190}	{191}	{191}	{190}
SF-36	−0.351^*∗∗*^	−0.062	−0.300^*∗∗*^	0.253^*∗∗*^	−0.150^*∗*^	0.423^*∗∗*^
Total score	{190}	{186}	{187}	{188}	{189}	{187}
EQ-5D	−0.242^*∗∗*^	0.015	−0.193^*∗∗*^	0.086	−0.177^*∗*^	0.085
VAS	{204}	{200}	{202}	{202}	{202}	{199}
CLDQ	−0.211^*∗∗*^	−0.017	−0.090	0.019	−0.026	0.237^*∗∗*^
Full scale	{153}	{153}	{153}	{153}	{153}	{153}

^*∗*^ *P* < 0.05 and ^*∗∗*^*p* < 0.01 correlational relationship.

## Data Availability

The data used in this article are available from the corresponding author upon reasonable request (ljli@zju.edu.cn).

## Supplementary Materials

Supplementary Materials Supplementary Table 1: the laboratory parameters were obtained from medical records or the hospital database. The CHB and HB cirrhosis groups were compared according to the baseline laboratory parameters. A normal distribution test indicated whether the data were parametric. Total bilirubin met normal distribution and was analyzed using Student's *t*-test. Other data were analyzed using Mann–Whitney *U* tests to compare the two HB groups. Supplementary Figure 1: we briefly summarized the incorporation process of patients with HB, including patients with CHB and HB cirrhosis. Three hundred potential patients with HB participated in this study. Among these 300 patients, 146 patients were excluded for not receiving antiviral therapy, leaving 164 patients with HB. Ten patients were excluded for returning incomplete questionnaires.
