# Supplementary material for: Health-Related Quality of Life and Its Influencing Factors in Patients with Hepatitis B: A Cross-Sectional Assessment in Southeastern China
Source: Can J Gastroenterol Hepatol. 2021 Jul 7;2021:9937591. doi: 10.1155/2021/9937591 (PMC8279869; doi:10.1155/2021/9937591)
Supplement: Supplementary Materials — Supplementary Table 1: the laboratory parameters were obtained from medical records or the hospital database. The CHB and HB cirrhosis groups were compared according to the baseline laboratory parameters. A normal distribution test indicated whether the data were parametric. Total bilirubin met normal distribution and was analyzed using Student's t-test. Other data were analyzed using Mann–Whitney U tests to compare the two HB groups. Supplementary Figure 1: we briefly summarized the incorporation process of patients with HB, including patients with CHB and HB cirrhosis. Three hundred potential patients with HB participated in this study. Among these 300 patients, 146 patients were excluded for not receiving antiviral therapy, leaving 164 patients with HB. Ten patients were excluded for returning incomplete questionnaires. [file 9937591.f1.zip › 9937591.f1/supp figure (1).docx]

Health-related quality of life and the factors that influence it in patients with hepatitis B: A cross-sectional assessment in southeastern China

**Supplemental Figure 1.** Patient enrollment flow.

300 HB patients

164 HB patients

Inclusion and exclusion criteria

154 HB patients

10 patients left more than one item incomplete
