# Supplementary material for: Health-Related Quality of Life and Its Influencing Factors in Patients with Hepatitis B: A Cross-Sectional Assessment in Southeastern China
Source: Can J Gastroenterol Hepatol. 2021 Jul 7;2021:9937591. doi: 10.1155/2021/9937591 (PMC8279869; doi:10.1155/2021/9937591)
Supplement: Supplementary Materials — Supplementary Table 1: the laboratory parameters were obtained from medical records or the hospital database. The CHB and HB cirrhosis groups were compared according to the baseline laboratory parameters. A normal distribution test indicated whether the data were parametric. Total bilirubin met normal distribution and was analyzed using Student's t-test. Other data were analyzed using Mann–Whitney U tests to compare the two HB groups. Supplementary Figure 1: we briefly summarized the incorporation process of patients with HB, including patients with CHB and HB cirrhosis. Three hundred potential patients with HB participated in this study. Among these 300 patients, 146 patients were excluded for not receiving antiviral therapy, leaving 164 patients with HB. Ten patients were excluded for returning incomplete questionnaires. [file 9937591.f1.zip › 9937591.f1/supp table.docx]

Health-related quality of life and the factors that influence it in patients with hepatitis B: A cross-sectional assessment in southeastern China

**Supplementary materials**

| **Supplementary Table 1. Baseline characteristics of chronic hepatitis B virus infection patients.** | | | | |
| --- | --- | --- | --- | --- |
| **Laboratory parameters** | **Entire cohort**  **N = 154** | **CHB**  **N = 98** | **HB cirrhosis**  **N = 56** | ***P*** |
| ALT, U/L | 32.95 (23.93, 43.05) | 30.60 (23.30, 45.00) | 33.00 (24.10, 41.00) | 0.854 |
| AST, U/L | 26.00 (21.00, 36.03) | 25.00 (20.00, 37.00) | 28.45 (24.00, 35.65) | 0.076 |
| TB, µmol/L | 17.83 ± 7.82 | 17.16 ± 7.67 | 19.00 ± 8.02 | 0.160 |
| DB, µmol/L | 3.15 (2.20, 4.70) | 2.90 (2.20, 4.30) | 3.70 (2.45, 6.20) | 0.036 |
| Total protein, g/L | 77.00 (73.35, 80.45) | 77.00 (74.00, 80.00) | 78.00 (73.00, 81.00) | 0.732 |
| Albumin, g/L | 43.00 (41.00, 45.05) | 43.15 (42.00, 45.50) | 43.00 (39.30, 44.70) | 0.071 |
| GGT, U/L | 21.10 (14.05, 36.25) | 19.00 (12.90, 29.00) | 26.50 (18.80, 47.30) | 0.003 |
| ALP, U/L | 94.05 (73.15, 114.63) | 92.70 (73.00, 111.10) | 98.25 (77.50, 124.00) | 0.224 |
| AFP, ng/ml | 4.04 (2.78, 6.46) | 3.90 (2.76, 5.44) | 4.38 (2.88, 7.16) | 0.192 |
| Continuous variables are presented *as* *M* ± SD or median (25–75 percentile). ALP, alkaline phosphatase; ALT, alanine aminotransferase; AST, aspartate aminotransferase; TB, total bilirubin; DB, direct bilirubin; GGT, gamma‐glutamyl transpeptidase; AFP, alpha fetoprotein. | | | | |
